# Accuracy of vital sign monitoring using a photoplethysmography upper arm wearable device in postoperative non-cardiac surgery patients: a prospective observational clinical validation study

**DOI:** 10.1007/s10877-025-01358-z

**Published:** 2025-09-22

**Authors:** Noa Reijmers, Arthur van Kootwijk, Eric E. C. de Waal

**Affiliations:** 1 Department of Technical Medicine, Delft University of Technology, Delft, The Netherlands; 2 Department of Technical Medicine, Leiden University Medical Center, Leiden, The Netherlands; 3 Department of Technical Medicine, Erasmus University Medical Center, Rotterdam, The Netherlands; 4 Department of Computerization Automation and Medical Technology (iMED), OLVG, Amsterdam, The Netherlands; 5 Department of Anesthesiology, University Medical Center Utrecht, Utrecht, The Netherlands

**Keywords:** Remote patient monitoring, Wearable device, Photoplethysmography, Clinical deterioration, Vital signs

## Abstract

Vital sign monitoring in patients is essential for the early detection of deterioration of vital signs and timely medical intervention especially on general wards in hospitals. Traditionally performed manually and intermittently, wearable monitoring devices offer a promising alternative by automatically providing real-time, continuous data. In this prospective observational study in non-cardiac surgery patients, we aim to evaluate the accuracy of respiratory rate (RR), heart rate (HR), and peripheral oxygen saturation (SpO₂) measurements obtained from a photoplethysmography (PPG)-based upper arm wearable device viQtor® (smartQare, Eindhoven, The Netherlands), by simultaneously comparing its readings with standard monitoring equipment in the Post-Anesthesia Care Unit (PACU). Capnography was included as the gold-standard reference for RR. Agreement between the wearable and reference measurements were assessed using Bland–Altman analyses. Clinical accuracy was evaluated using Clarke Error Grid analyses. Vital sign data were collected from 42 postoperative patients (age: 65.5 years [IQR 37.4–74.7]; BMI: 24.1 kg/m^2^ [IQR 21.7–26.9]) over a median duration of 14.0 hours. The Average Root Mean Square (ARMS) between the wearable device and the reference for RR was 2.85 BRPM, with a bias of -0.40 (95% LoA –5.85 to 5.04); for HR 2.01 BPM, with a bias of 0.08 (95% LoA -3.83 to 3.99); and for SpO_2_ 2.08%, with a bias of –0.03 (95% LoA –4.14 to 4.09). The viQtor® device demonstrated high accuracy for RR, HR, and SpO₂ in postoperative patients. Data availability was high across all three parameters, and patient satisfaction was excellent. These findings support its potential for continuous monitoring on general wards.

**Supplementary Information:**

The online version contains supplementary material available at 10.1007/s10877-025-01358-z.

## Introduction

Vital signs, including respiratory rate (RR), heart rate (HR), and oxygen saturation (SpO_2_), are fundamental indicators of a patients’ physiological status and are crucial for early detection of clinical deterioration [1–4]. On general hospital wards however, these parameters are still typically measured manually and intermittently by nursing staff, most often at 8-hour intervals. This approach increases the risk of delayed recognition of clinical deterioration and imposes a substantial workload on clinical staff [5–7].

Wearable monitoring devices offer a promising solution by enabling continuous, non-invasive and wireless monitoring of vital signs [8–10]. Clinical studies have shown that continuous monitoring facilitates earlier intervention, lowers complication rates, reduces intensive care unit (ICU) admissions, shortens hospital stay, and decreases both nursing workload and overall healthcare costs [6, 11–17].

Despite their potential, the clinical accuracy of wearable monitoring devices is insufficiently evaluated, especially for multi-parameter systems that include SpO₂. Most validation studies to date have focused solely on HR [18–20], or HR and RR [21–23], while SpO_2_ is frequently not included in devices under investigation, despite evidence that up to 80% of desaturation episodes remain undetected with standard intermittent monitoring [7].

Only a limited number of studies have evaluated wearable medical devices capable of simultaneously measuring RR, HR, and SpO_2_ in clinical settings [24, 25]. One such study assessed a device that relied on multiple wired components which limits its usability in clinical environments, especially in lower-acuity settings such as general wards and home-based monitoring [25]. Another study evaluated a fully wireless wearable medical device, but data collection was restricted to a brief 40-minutes monitoring period [24].

The goal of our study is to clinically validate the viQtor® wireless wearable medical device (smartQare, Eindhoven, The Netherlands) for continuous monitoring of RR, HR, and SpO_2_ in postoperative patients. This population is particularly vulnerable to respiratory and hemodynamic complications and fluctuations in vital signs [26–28]. In contrast to previous studies, this study includes all three vital parameters and evaluates accuracy over extended monitoring periods, providing a more comprehensive and clinically relevant assessment of device performance. Additionally, we evaluate patient-reported satisfaction with the device worn on one of the upper arms.

## Methods

This prospective observational clinical validation study was conducted in the Post-Anesthesia Care Unit (PACU) in the University Medical Center Utrecht (UMCU), Utrecht, The Netherlands. The study was conducted following Good Clinical Practice and was performed in accordance with the ethical standards as laid down in the 2002 Declaration of Helsinki. Formal ethical approval was obtained from the Medical Research Ethics Committee of the UMCU, Utrecht, The Netherlands (METC 23–240).

### Study population

Included were adult patients (≥ 18 years) who were expected to be admitted to the PACU for at least 6 hours after elective major non-cardiac surgery. Excluded were patients with known skin hypersensitivity, allergic reactions to metals or plastics, tattoos at the intended sensor placement site, significant upper arm deformities or infections, compromised upper arm blood flow, presence of tremors or convulsions, or upper arm circumferences exceeding the device’s fitting range (> 43 cm). Patients who agreed to participate signed the informed consent form prior to surgery.

### Study procedure

Immediately after surgery at admission to the PACU, the viQtor® wearable device (firmware version 2.2.0) was applied on one of the patient’s upper arms to start continuous measurement of RR, HR, and SpO_2_. Simultaneously, standard of care bedside monitoring was conducted using the Spacelab XPREZZON® 91393 (Spacelab Healthcare, Snoqualmie, USA). Patients continued to be monitored with both systems until the next morning, up to a maximum of 24 hours.

Upon completion of the monitoring period or upon transfer to the general ward, participants were asked to complete a brief satisfaction survey. This survey included three statements assessed on a 5-point Likert scale (1 = strongly disagree, 2 = disagree, 3 = neutral, 4 = agree, 5 = strongly agree):

1. I experienced the device as comfortable.
2. I would be willing to wear the device again during a next hospital stay.
3. I experienced skin reactions during or after wearing the armband with the device.

### Investigational device (viQtor®)

**Fig. 1:**
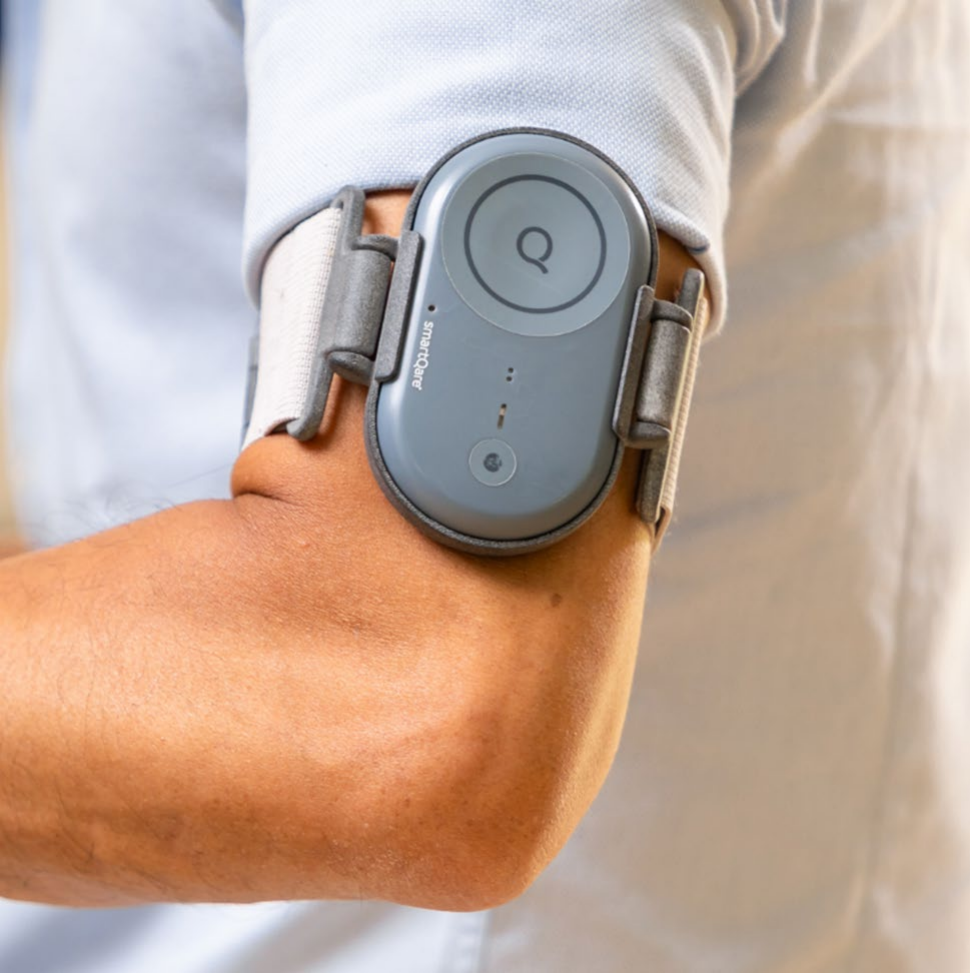
The viQtor® wearable medical device (smartQare, Eindhoven, The Netherlands). The wearable sensor, attached to the upper arm, measures RR, HR, and SpO_2_ using PPG signals. Additional features include skin temperature, activity index, and fall detection, which were not evaluated in this study

The investigational device, viQtor® is a CE-certified wearable monitoring system designed to be worn on the upper arm (Fig. 1). It uses photoplethysmography (PPG) technology to continuously monitor RR, HR, and SpO_2_. The device also provides non-vital parameters, including skin temperature, an activity index, and fall detection. However, these last three features were not evaluated in this study. viQtor® is reusable and equipped with a rechargeable battery, offering an average operational duration of 5 days per fully charged battery.

For research purposes, data containing one-minute average values (mean of each 60-second interval) per parameter were stored on a SD card for offline analysis.

In clinical use, viQtor® can wirelessly transmit the one-minute average values in batches every 5 minutes via a secure mobile network (LTE-M/NB-IoT, part of the 4G/5G infrastructure). The device sends the information directly to a secure cloud platform without requiring connection to a smartphone or additional application. Data can then be accessed through a web-based interface or integrated into the electronic health record (EHR). These functionalities were not used or tested in this study.

### Reference bedside monitor

The XPREZZON® 91393 is a clinically validated bedside monitor equipped with cables and disposables to continuously track multiple vital signs, including RR, HR, SpO_2_, blood pressure, and body temperature. HR is derived from ECG and SpO_2_ is measured using pulse oximetry at the fingertip. Capnography was used as reference technique to ensure adequate RR measurements. The Smart Advanced Capnoline® H Plus EtCO_2_ sampling line (Medtronic, Boulder, USA) was connected to the Spacelab monitor only during the initial hours of monitoring to minimize patient burden.

### Signal analysis

Data from the viQtor® and reference systems were processed using Python 3.10. Signals were synchronized by maximizing signal correlation. Low quality data points from the viQtor® were automatically excluded based on its integrated quality index, which accounts for factors such as motion artifacts and low signal-to-noise ratios. For the reference ECG and capnography data, one-minute averages were computed to match the same time intervals of the viQtor® data. For SpO_2_, the reference monitor provided only one data point per minute, which was linearly interpolated to align with the exact time points of the viQtor® measurements.

### Statistical analysis

Statistical analysis was performed using Python 3.10. RR, HR and SpO_2_ were evaluated using Bland–Altman analysis for repeated measurements [29]. The primary outcome is the Average Root Mean Square (ARMS), accompanied by the bias and 95% limits of agreement (LoA) between viQtor® and the reference. To assess clinical acceptability, predefined ARMS thresholds were applied, derived from values commonly used in literature and international standards [30, 31]: RR ARMS ≤ 3 breaths/min (BRPM); HR ARMS ≤ 3 beats/min (BPM); and SpO_2_ ARMS ≤ 3%. Secondary outcomes included Clarke Error Grid analyses of RR and HR to evaluate the impact of measurement errors on clinical decision-making [32]. The Clarke Error Grid is a scatterplot-based method that categorizes data points into five regions (A-E) based on clinical relevance. Region A includes measurements within 20% of the reference. Region B includes measurements outside region A that would not lead to unnecessary treatment. Region C includes measurements that could result in unnecessary treatment. Region D represents potentially dangerous failures to detect a critical event (e.g. bradycardia, tachypnea), and region E reflects measurements where events are confusing (e.g., bradypnea with tachypnea). Thresholds for clinical relevance were based on the Modified Early Warning Score (MEWS) [33], with normal value ranges defined as 8–20 BRPM for RR and 40–100 BPM for HR. The exact region boundaries allow for some variation beyond these ranges, following standard Clarke Error Grid conventions [21, 34, 35]. Additionally, viQtor® data availability was assessed by calculating average data loss. Finally, patient satisfaction surveys were analyzed descriptively. Supplementary materials include individual error plots and Bland–Altman analyses comparing RR measurements between viQtor® and thoracic impedance pneumography, as well as capnography and thoracic impedance pneumography.

## Results

From January 2025 to May 2025, a total of 45 postoperative patients were initially included in the study. However, three patients were excluded due to incomplete data: two had no reference measurements available, and one had missing viQtor® data due to an incorrect start of the recording. The characteristics of the remaining 42 patients are summarized in Table 1.

In total, patients wore the viQtor® for 522 h, with a median duration of 14.0 h per patient (range 2.7–22.3 h). Specifically, monitoring with the capnography sampling line was done for 341 h, with a median duration of 6.2 h per patient (range 2.4–19.9 h).

**Table 1:** Patient characteristics (n = 42)

Female, n (%)	22 (52.4)
Age (years), median [IQR]	65.5 [37.4–74.7]
Height (m), median [IQR]	1.73 [1.68–1.85]
Weight (kg), median [IQR]	73 [64–82]
BMI (kg/m^2^), median [IQR]	24.1 [21.7–26.9]
Surgical subspecialty, n (%)	
Neurosurgery	26 (61.9)
Abdominal surgery	10 (23.8)
Vascular surgery	5 (11.9)
Head and neck surgery	1 (2.4)
Comorbidities, n (%)	
Heart disease (ischemic, valvular, arrhythmias)	6 (14.3)
Hypertension	3 (7.1)
Peripheral vascular disease	2 (4.8)
Cerebrovascular disease	1 (2.4)
Lung disease (COPD, asthma, fibrosis)	8 (20.0)
OSAS	1 (2.4)
ASA physical status, median [IQR]	2.5 [, 2–3]
Monitoring duration viQtor® (hours), median [IQR]	14.0 [5.7–18.4]
Monitoring duration capnography (hours), median [IQR]	6.2 [5.1–9.6]

ASA physical status, American Society of Anesthesiologists Physical Status Classification System; BMI, Body Mass Index; COPD, Chronic Obstructive Pulmonary Disease; IQR, Interquartile Range; kg, kilograms; m, meter; n, number of patients; OSAS, Obstructive Sleep Apnea Syndrome

### Respiratory rate

**Fig. 2:**
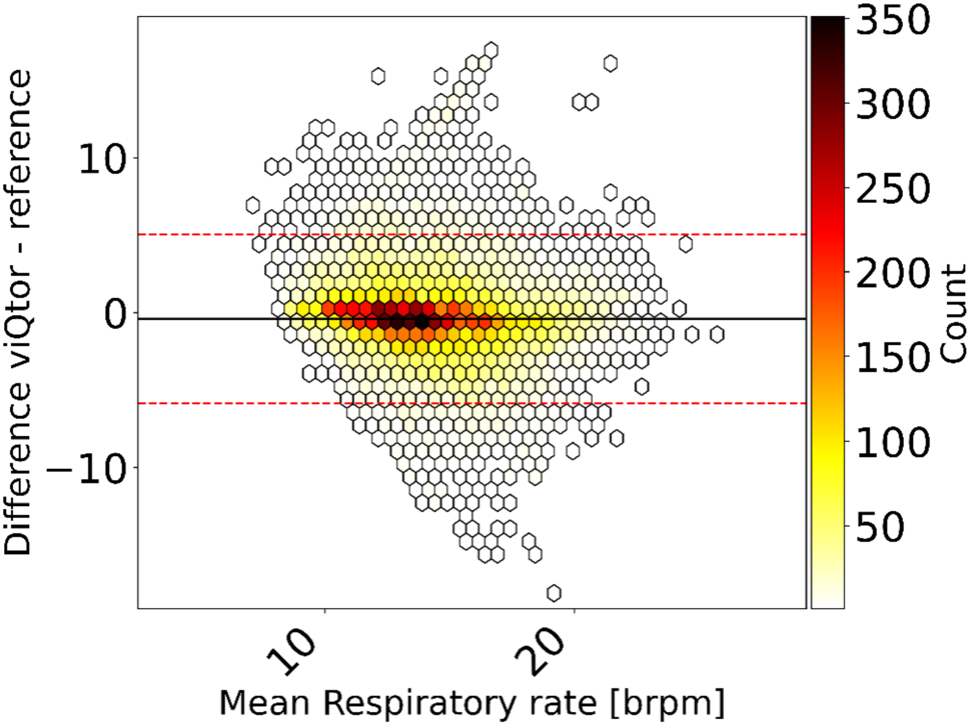
Bland–Altman plot from the pooled analysis comparing viQtor® respiratory rate measurements to the capnography reference, with color indicating the number of measurement pairs (white = low, black = high). The solid black line represents the bias and the dashed red line the limits of agreement

A total of 17,425 RR measurement pairs were available for analysis. Data availability from the viQtor® was 95.4%. The overall ARMS was 2.85 BRPM, with a bias of –0.40 BRPM and LoA of –5.85 to 5.04 BRPM (Fig. 2, Table 2). These results remained below the predefined acceptable threshold. Fig. S1a (Supplement 1) shows the error plot of individual results.

**Table 2:** Accuracy outcomes for all three vital signs measured by the viQtor® compared to the reference monitors

	Number of data pairs	ARMS	Bias	Lower 95% LoA	Upper 95% LoA
RR	17,425	2.85	−0.40	−5.85	5.04
HR	27,361	2.01	0.08	−3.83	3.99
SpO_2_	26,842	2.08	−0.03	−4.14	4.09

ARMS, Average Root Mean Square; HR, Heart Rate; LoA, Limits of Agreement; RR, Respiratory Rate; SpO_2_, peripheral oxygen saturation

### Heart rate

**Fig. 3:**
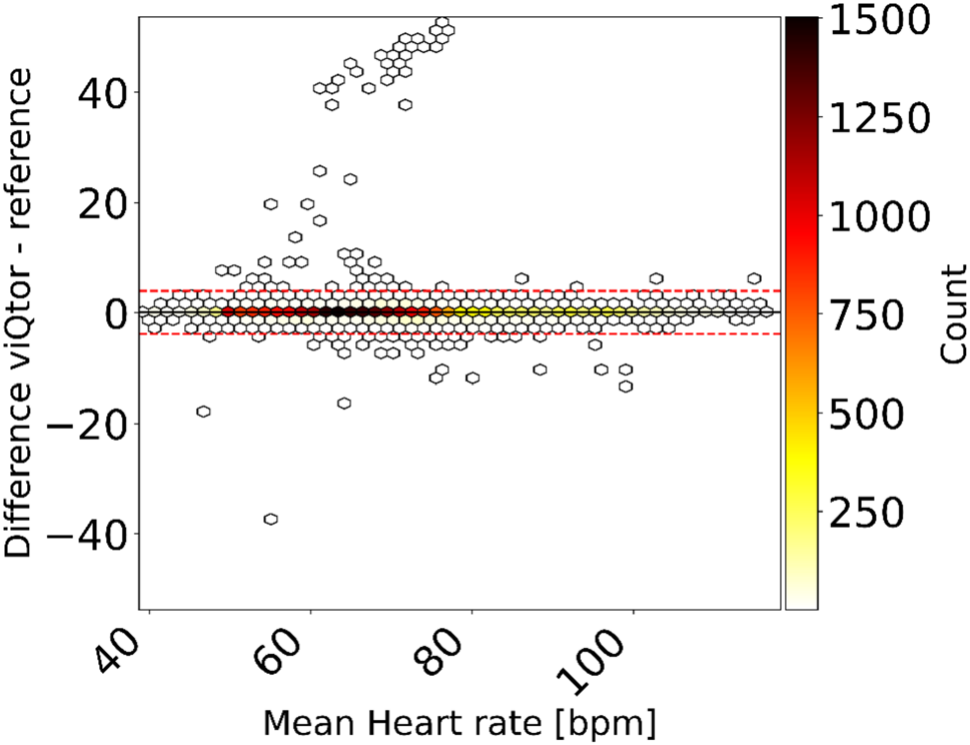
Bland–Altman plot from the pooled analysis comparing viQtor® heart rate measurements to the ECG-derived reference, with color indicating the number of measurement pairs (white = low, black = high). The solid black line represents the bias and the dashed red line the limits of agreement

A total of 27,361 HR measurement pairs were available for analysis. Data availability from the viQtor® was 98.7%. The overall ARMS was 2.01 BRPM, with a bias of 0.08 BRPM and narrow LoA of –3.83 to 3.99 BRPM (Fig. 3, Table 2). These results remained below the predefined acceptable threshold. Fig. S1b (Supplement 1) shows the error plot of individual results.

### Oxygen saturation

**Fig. 4:**
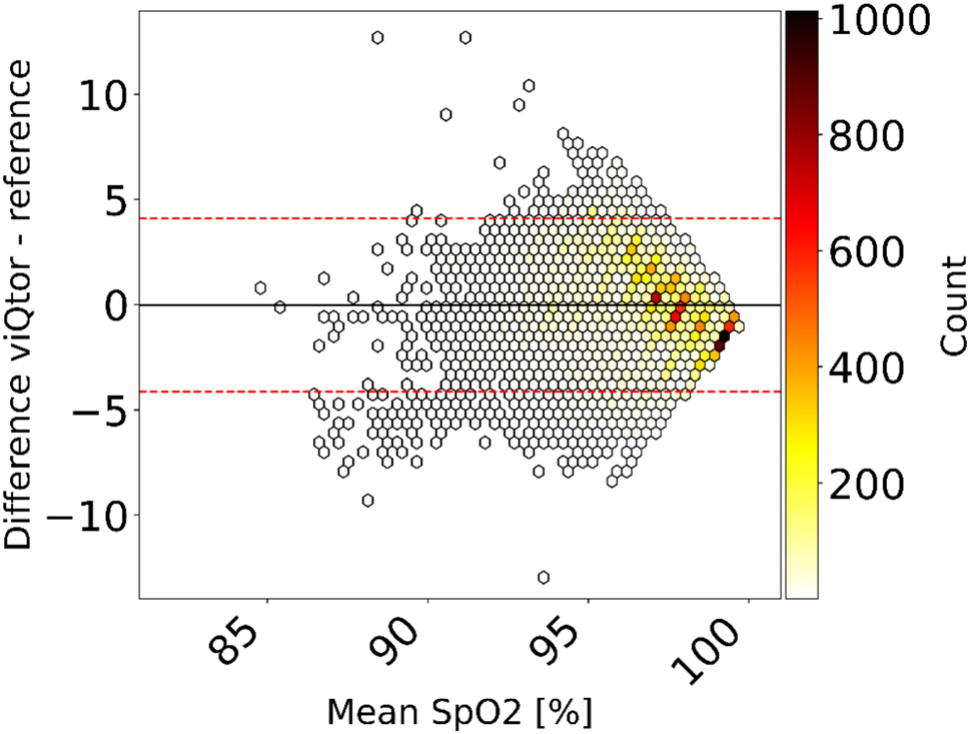
Bland–Altman plot from the pooled analysis comparing viQtor® SpO_2_ measurements to the reference pulse oximeter, with color indicating the number of measurement pairs (white = low, black = high). The solid black line represents the bias and the dashed red line the limits of agreement

A total of 26,842 SpO_2_ measurement pairs were available for analysis. Data availability from the viQtor® was 90.6%. The overall ARMS was 2.08%, with a bias of −0.03% and LoA of –4.14 to 4.09% (Fig. 4, Table 2). These results remained below the predefined acceptable threshold of ≤ 3%. Fig. S1c (Supplement 1) shows the error plot of individual results.

### Clarke error grid analysis

**Fig. 5:**
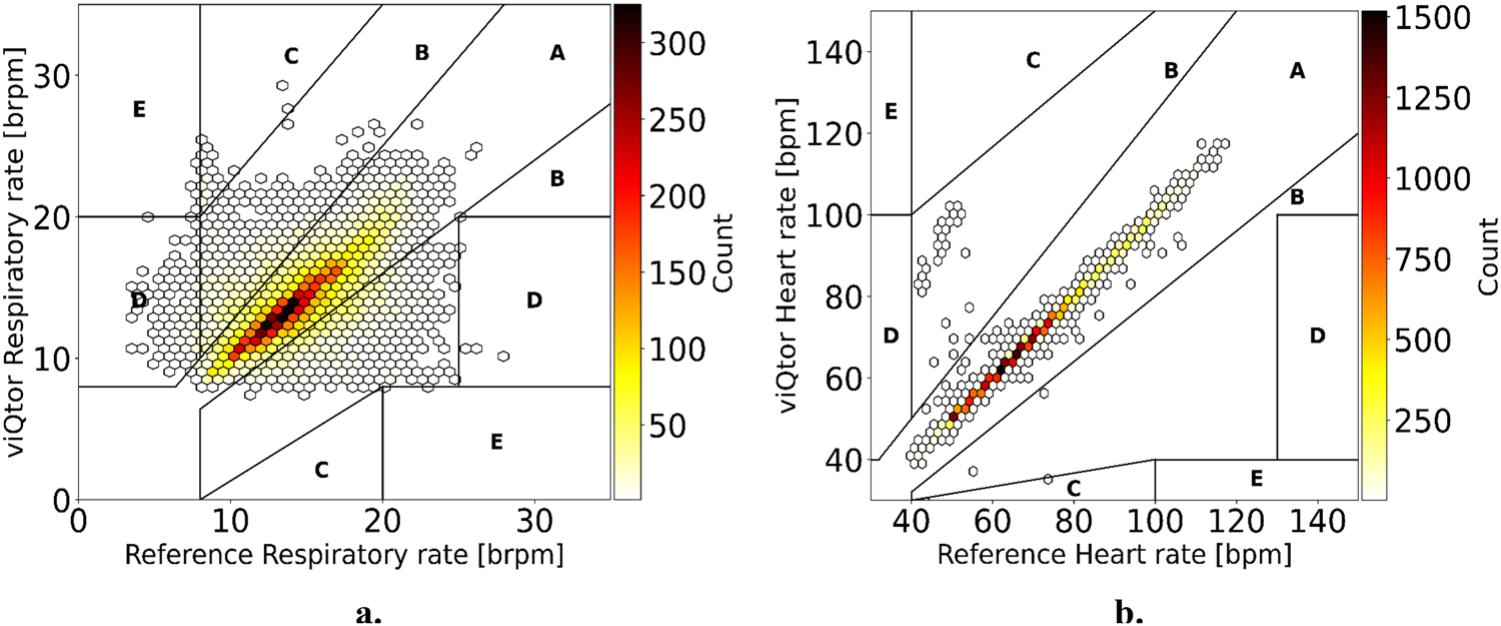
Clarke Error Grid analysis of (**a**): respiratory rate and (**b**): heart rate measurements, with color indicating the number of measurement pairs (white = low, black = high). Region A includes measurements within 20% of the reference. Region B includes measurements outside region A that would not lead to unnecessary treatment. Region C includes measurements that could result in unnecessary treatment. Region D represents potentially dangerous failures to detect a critical event (e.g. bradycardia, bradypnea), and region E reflects measurements where events are confusing (e.g., bradypnea with tachypnea)

The Clarke Error Grid analysis for RR and HR are presented in Fig. 5, with the distribution of data pairs across regions A to E summarized in Table 3. For RR, 98.4% of measurements fell within regions A or B, indicating that the device would support appropriate clinical decision-making in the vast majority of cases. Only 1.6% of RR values were located in regions C, D, or E, suggesting minimal risk of unnecessary interventions, missed treatments, or misinterpretation of critical conditions (e.g., confusing bradypnea with tachypnea). For HR, 100% of measurements were classified within region A or B, demonstrating excellent clinical accuracy of the wearable device.

**Table 3:** Clarke error grid analysis outcomes for respiratory rate and heart rate

	**Region A,** **N (%)**	**Region B,** **N (%)**	**Region C,** **N (%)**	**Region D,** **N (%)**	**Region E,** **N (%)**
Respiratory rate	78.9	19.5	0.4	1.2	0.1
Heart rate	99.8	0.2	0	0	0

N, Number of measurement pairs

### Patient satisfaction

Patients responded very positively to the device. On the 5-point Likert scale survey (1 = strongly disagree, 5 = strongly agree), 98% rated the device as comfortable (n = 40, score 5; n = 1, score 4) and were willing to wear it again (n = 38, score 5; n = 3, score 4). Only one patient (2%) selected the lowest score for both statements, which may reflect a misunderstanding of the scale rather than actual dissatisfaction. No skin reactions were reported.

## Discussion

### Principal findings

This study evaluated the performance of the upper arm PPG-based wearable device (viQtor®) for continuous monitoring of RR, HR, and SpO_2_ in a cohort of postoperative patients (ASA physical status median 2.5 [IQR 2–3]) with some having cardiopulmonary comorbidities, such as cardiac arrhythmias treated with cardiac pacemakers, and COPD (Table 1). This diversity ensured a broad spectrum of patients and comorbidities, contributing to a robust and clinically relevant validation process.

The device showed high agreement with gold-standard reference methods for RR and HR and remained well within the acceptability threshold for SpO_2_ compared to the reference pulse oximeter. Data availability was high across all vital signs, and patients found the device comfortable and were willing to wear it again.

Agreement for RR was high when compared to the gold-standard capnography, with an ARMS ≤ 3 BRPM. In contrast, comparison with thoracic impedance pneumography yielded substantially lower agreement (ARMS = 4.98 BRPM; Fig S2a, Supplement 2), highlighting the impact of an adequate reference method. While impedance pneumography is widely used for continuous RR monitoring in PACU settings, it is prone to inaccurate measurements due to motion artifacts, ECG sticker detachment, and speech interference [36]. A direct comparison between capnography and thoracic impedance pneumography yielded unacceptable agreement (ARMS = 5.39 BRPM; Fig. S2b, Supplement 2), emphasizing the limitations of impedance-based RR monitoring and the need for robust reference methods in validation studies. These findings also reflect the current challenge of accurately measuring RR in clinical practice.

HR measurements from the viQtor® wearable showed high agreement with the reference monitor and excellent clinical accuracy. However, one outlier patient had an ARMS of 10 BPM (Fig. S1b, Supplement 1) which was attributed to periods of erroneously high HR values caused by poor PPG signal quality. This was likely due to low perfusion at the sensor site, possibly resulting from the patient lying on the arm where the device was worn.

SpO_2_ measurements from the viQtor® wearable also showed high agreement with the reference pulse oximeter, even though the reference was not a gold-standard. In the example data shown in Fig. S3 (Supplement 3), the viQtor® device reported slightly lower SpO₂ values compared to the reference, which measured prolonged readings of 100% saturation. This discrepancy does not reflect overall results, as the pooled Bland–Altman analysis confirmed the absence of systematic bias between the two devices (Fig. 4).

In contrast to previous validation studies, which focused on fewer vital signs or brief monitoring periods (30 to 40 minutes) [18–25], our study continuously evaluated three key parameters (RR, HR, and SpO_2_) over a median period of 14 hours per patient, providing a more robust assessment of device performance throughout the PACU stay (Supplement 3: Example of vital signs trend data in one patient). Similarly, Breteler et al. recently conducted a validation study of a multi-parameter wearable (Checkpoint Cardio system) with a median monitoring duration of 26 hours in surgical wards. They reported a respiratory rate bias of 1.5 BRPM (LoA −3.7 to 7.5), HR bias of 0.0 BPM (LoA −3.5 to 3.4), and SpO_2_ bias of 0.4% (LoA −3.1 to 4.0) [35], which are comparable to our results. However, the Checkpoint Cardio system is considerably more complex and intrusive, consisting of multiple wired components. Qualitative studies have shown that cumbersome or intrusive devices reduce acceptance among both patients and nurses, hindering clinical implementation [37–39]. In our study, 98% of patients rated the viQtor® as comfortable and expressed willingness to wear it again. Comparative data remain limited, particularly for upper arm worn devices. For example, Lockhorst et al. reported positive experiences in 69% of 191 patients using an adhesive patch sensor [38]. The higher satisfaction in our study may reflect design advantages of the viQtor®, which uses a soft, elastic arm band instead of adhesives. This design facilitates easy removal, repositioning, and minimizes the risk of skin irritation, features that support prolonged patient use and ease of use by nurses.

### Limitations

Several limitations should be considered. Although the study captured important variations in vital signs, the full physiological range was not represented, which may limit the generalizability of the observed device performance. Additionally, patient mobility was relatively low during monitoring in the PACU, whereas patients in general wards or ambulatory settings typically exhibit higher levels of physical activity. The viQtor® device incorporates a signal quality index that excludes segments with poor signal quality, which may be caused by motion artifacts. While this feature improves the accuracy and reliability of reported values, it may reduce data continuity in highly mobile populations. Nevertheless, given the high data availability observed in this study (95.4% for RR, 98.7% for HR, and 90.6% for SpO₂), it is reasonable to assume the device would still outperform standard intermittent monitoring practices in terms of data frequency and the potential for earlier detection of clinical deterioration, even under motion conditions.

Furthermore, SpO₂ measurements were compared against a pulse oximeter rather than arterial blood gas analysis (the gold-standard for oxygen saturation) which limits the robustness of the validation. However, given that the viQtor® device met the acceptable accuracy threshold in this comparison, it is reasonable that it would also meet this threshold when validated against the gold-standard.

### Future directions

Continuous remote vital sign monitoring has the potential to improve patient monitoring (and subsequently patient outcomes) and to reduce clinical workload, especially on general wards where high-risk patients may deteriorate between intermittent checks [40]. Several studies have reported significant reductions in ICU admissions [15], complication rates [41], length of stay [13, 15], and nurse workload [17]. Despite these promising findings, robust evidence remains limited.

To advance the field, future research should evaluate comprehensive implementation strategies that integrate continuous monitoring with deterioration detection algorithms, response protocols, and outcome measures reflecting the full clinical pathway [40, 42]. A prospective implementation study is currently underway in a surgical ward in the Netherlands [43]. Similar studies across diverse ward settings and patient populations are needed to optimize continuous monitoring strategies and clinical workflows. Attention should be given to minimizing alarm burden through context-sensitive alerting [44, 45] or trend-based assessments without real-time alarms [46].

## Conclusions

The PPG-based, upper arm–worn viQtor® device demonstrated high accuracy in measuring RR and HR compared to gold-standard references and met the acceptability threshold for SpO_2_ compared to a pulse oximeter. These results support viQtor®’s ability to accurately and continuously monitor postoperative patients at possible risk of clinical deterioration. Data availability was consistently high across all three parameters, and patient satisfaction was excellent. Together, these findings show the potential of the viQtor® device for continuous monitoring on general wards.

## Supplementary Information

Below is the link to the electronic supplementary material.

Supplementary file1 (DOCX 968 KB)

Supplementary file2 (DOCX 465 KB)

Supplementary file3 (DOCX 214 KB)

## Data Availability

All data generated and analyzed during this study will be made available by the corresponding author on reasonable request (after anonymization).
