# Supplementary material for: Accuracy of vital sign monitoring using a photoplethysmography upper arm wearable device in postoperative non-cardiac surgery patients: a prospective observational clinical validation study
Source: J Clin Monit Comput. 2025 Sep 22;40(2):557–65. doi: 10.1007/s10877-025-01358-z (PMC13053423; doi:10.1007/s10877-025-01358-z)
Supplement: Supplementary file 1 — Supplementary file1 (DOCX 968 KB) [file 10877_2025_1358_MOESM1_ESM.docx]

**Supplement 1 – Error plots individual patients**


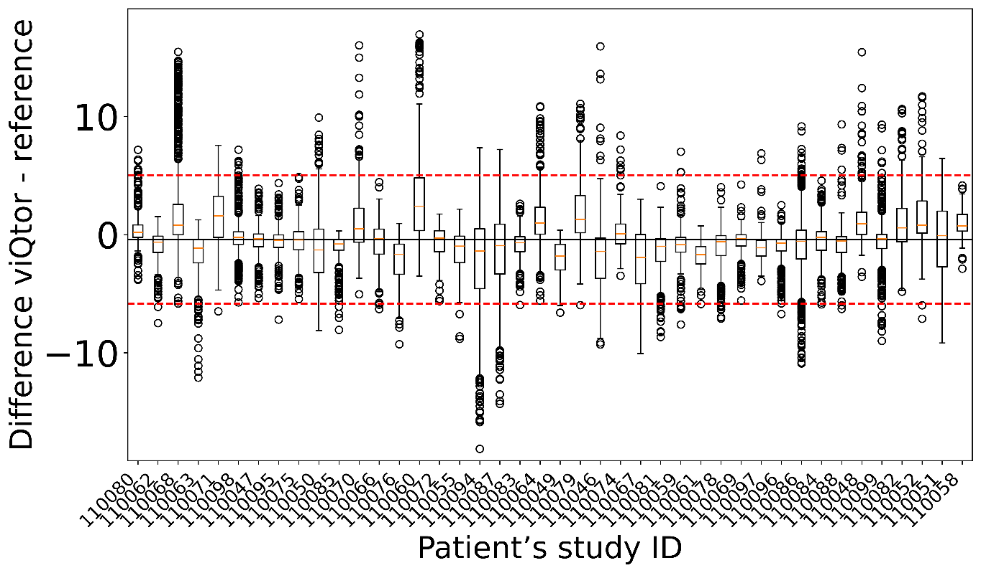

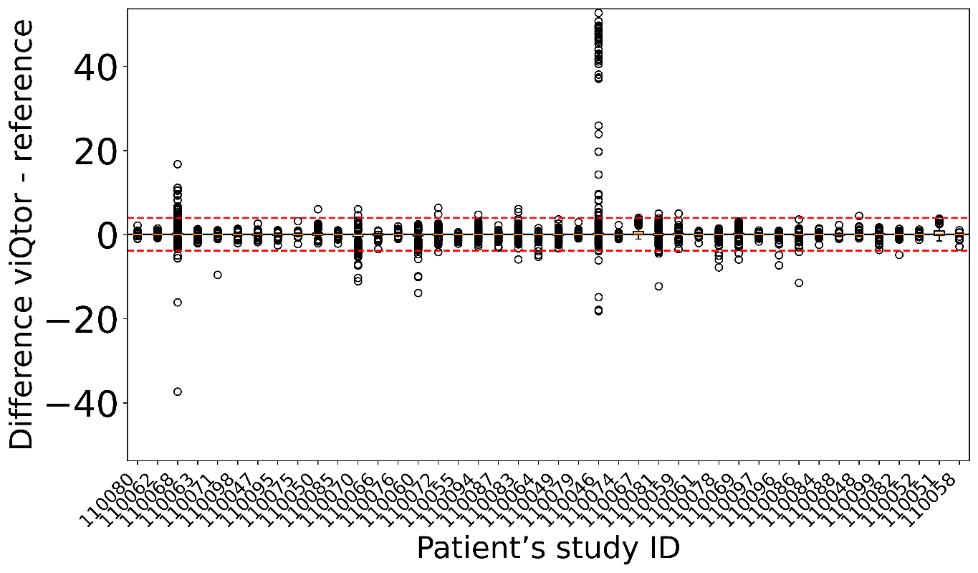

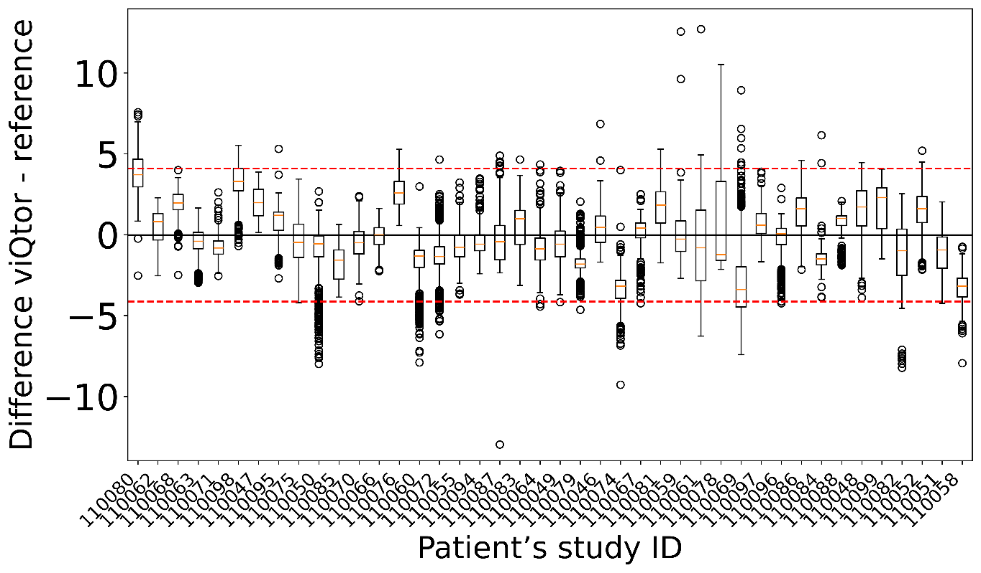


**a.**

**b.**

**c.**

**Fig. S1** Error plots of individual patient results comparing viQtor® measurements to the reference monitor. (a): Respiratory rate (capnography reference), (b): Heart rate, and (c): SpO_2_. The solid black line represents the pooled bias and the dashed red line the pooled limits of agreement
