# Supplementary material for: Accuracy of vital sign monitoring using a photoplethysmography upper arm wearable device in postoperative non-cardiac surgery patients: a prospective observational clinical validation study
Source: J Clin Monit Comput. 2025 Sep 22;40(2):557–65. doi: 10.1007/s10877-025-01358-z (PMC13053423; doi:10.1007/s10877-025-01358-z)
Supplement: Supplementary file 2 — Supplementary file2 (DOCX 465 KB) [file 10877_2025_1358_MOESM2_ESM.docx]

**Supplement 2 – Thoracic impedance pneumography comparisons**

**Thoracic impedance pneumography reference data**

A total of 26,668 reference pneumography-viQtor® RR measurement pairs were available for analysis. The overall ARMS was 4.98 BRPM, with a bias of 0.18 BRPM and wide LoA of –9.69 to 10.04 BRPM (Fig. S2a). Additionally, a Bland-Altman analysis comparing capnography and thoracic impedance pneumography showed an overall ARMS of 5.39 BRPM, with a bias of 0.84 BRPM and wide LoA of -9.81 to 11.50 (Fig. S2b). For illustration purposes, Fig. S2c shows a representative example in which impedance deviations are clearly visible across all three RR modalities.


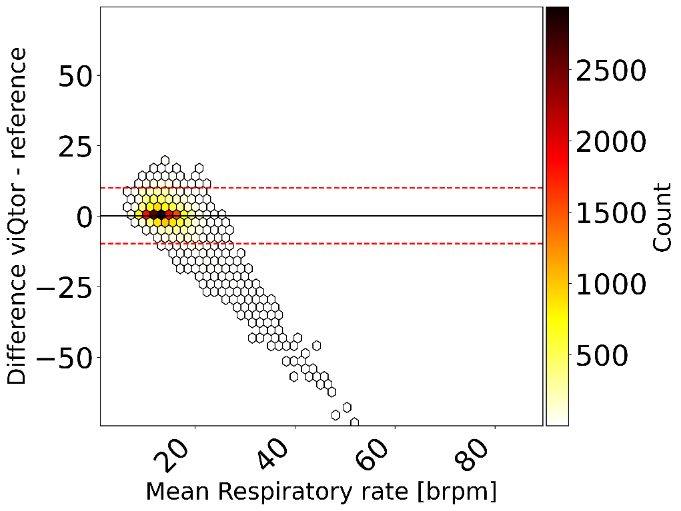

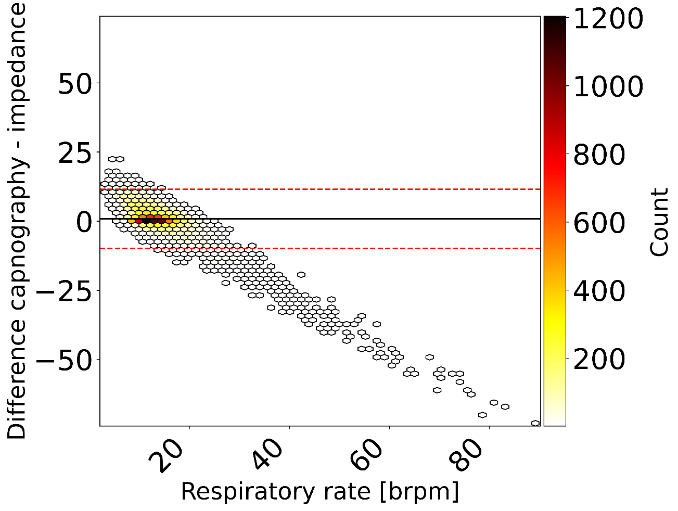


**a.**

**b.**

**Fig. S2** Bland-Altman plots of the pooled analysis comparing respiratory rate measurements from (a): viQtor and

thoracic impedance pneumography and (b): capnography and thoracic impedance pneumography, with color

indicating the number of measurement pairs (white = low, black = high). The solid black line represents the pooled

bias and the dashed red line the pooled limits of agreement.


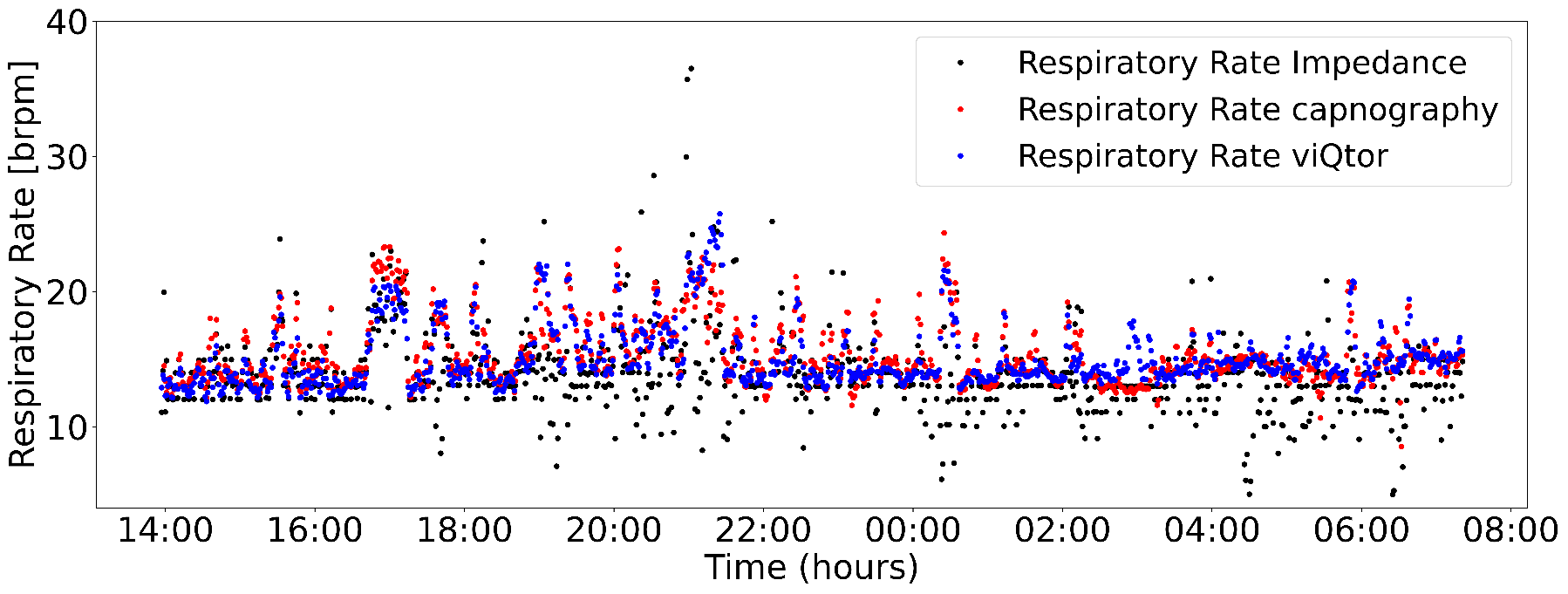


**Fig. S2c** Example of the difference between thoracic impedance pneumography-based (black), capnography-based (red), and viQtor® (blue) respiratory rate measurements
