# Supplementary material for: Accuracy of vital sign monitoring using a photoplethysmography upper arm wearable device in postoperative non-cardiac surgery patients: a prospective observational clinical validation study
Source: J Clin Monit Comput. 2025 Sep 22;40(2):557–65. doi: 10.1007/s10877-025-01358-z (PMC13053423; doi:10.1007/s10877-025-01358-z)
Supplement: Supplementary file 3 — Supplementary file3 (DOCX 214 KB) [file 10877_2025_1358_MOESM3_ESM.docx]

**Supplement 3 -** **Example of vital sign trend data**

Fig. S3 presents continuous vital sign data over a 22.3-hour period from one of the included postoperative patients, comparing the viQtor® with the reference monitor. To show the full 22-hour trends, pneumography was used as the reference for respiratory rate, as capnography was only available during the first 5 hours. The trends show a natural decline in both respiratory rate (RR) and heart rate (HR) during the initial hours following surgery, with lower values observed during the night. Notably, two sustained desaturation periods are visible in the SpO_2_ trend, highlighting viQtor®’s ability to capture clinically relevant fluctuations. These episodes may have been triggered by routine nursing care activities such as washing or repositioning.


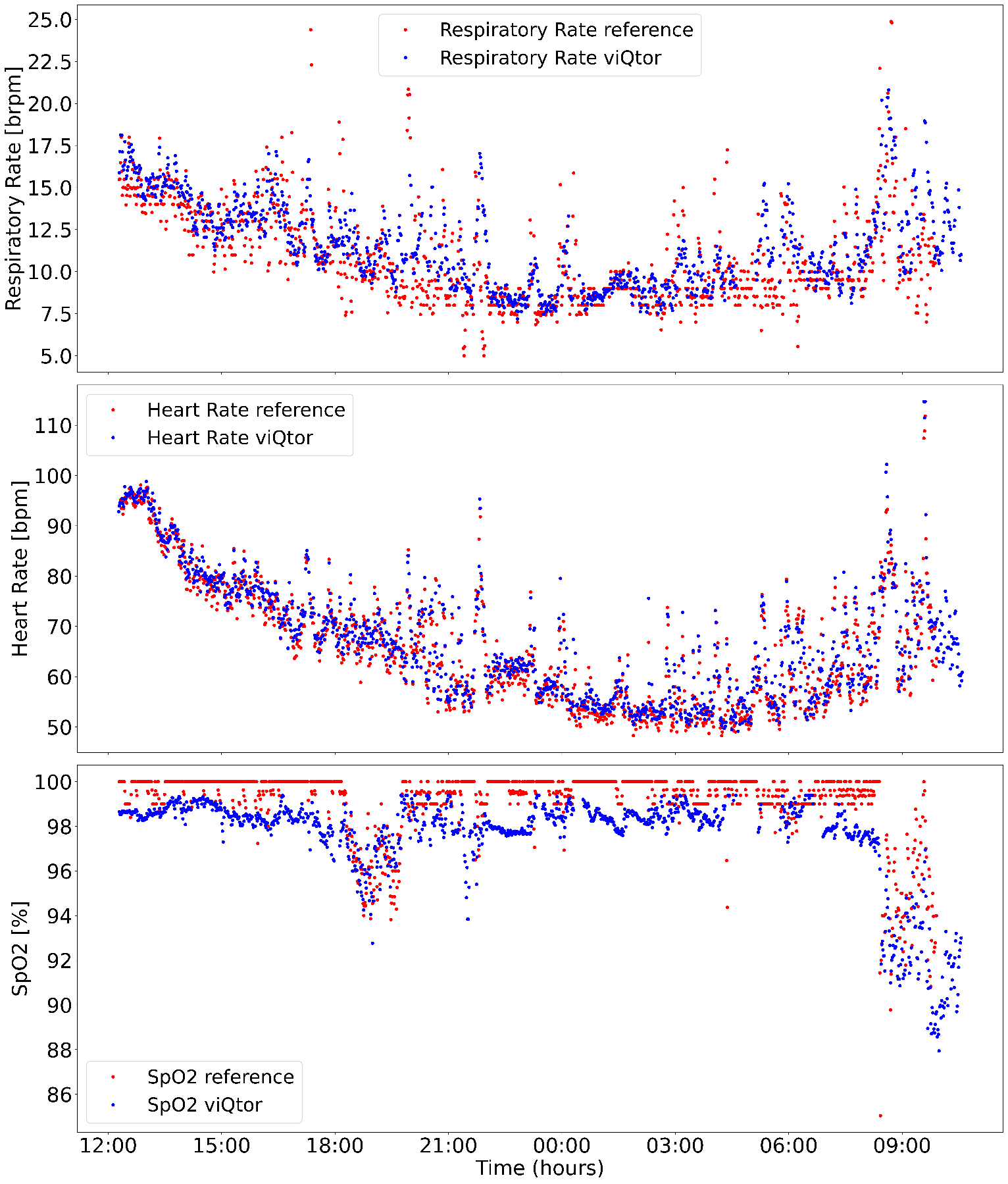


**Fig. S3** Example vital sign trend data from the viQtor® (blue) and reference monitor (red) during 22.3 hours of continuous monitoring, demonstrating variability in vital sign values. From top to bottom, the graphs show respiratory rate (pneumography reference), heart rate, and SpO_2_ measurements. Pneumography was used as the reference in this illustration because capnography was not available for the full monitoring period.
